# Occurrence and anti-microbial susceptibility pattern of extended spectrum beta-lactamase producing Enterobacteriaceae in governmental hospitals wastewater in Addis Ababa, Ethiopia

**DOI:** 10.1186/s41182-022-00437-0

**Published:** 2022-08-22

**Authors:** Alehegn Amare Kebede, Tesfaye Legesse Bedada, Dejenie Shiferaw Teklu, Degefu Beyene, Kassu Desta Tullu

**Affiliations:** 1 Department of Medical Laboratory Sciences, College of Health Sciences, Addis Ababa University, Addis Ababa, Ethiopia; 2 Department of Public Health Microbiology, Ethiopian Public Health Institute, Addis Ababa, Ethiopia; 3 Department of Bacteriology and Virology, Ethiopian Public Health Institute, Addis Ababa, Ethiopia

**Keywords:** Hospital, Wastewater, Beta lactamase, ESBLs, Enterobacteriaceae, Multi-drug resistance

## Abstract

**Background:**

Worldwide, come out and dissemination of extended-spectrum beta-lactamases (ESBLs) producing Enterobacteriaceae has been warning the efficacy of antibiotics to treat an infection. Hospital wastewaters were a reservoir of such kind of resistant bacteria. Currently, the predominant antibiotics used for the treatment of hospitalized patients infected by Gram-negative bacteria are the β-lactam antibiotics. Therefore, it is an important source to investigate the magnitude of ESBLs producing bacteria and their antimicrobial susceptibility pattern. This study aimed to determine the occurrence of ESBLs producing Enterobacteriaceae (ESBLs-pE) and their antibiotic susceptibility pattern in wastewater released from five governmental hospitals in Addis Ababa, Ethiopia.

**Methods:**

A cross-sectional study was conducted from April 1 to May 31, 2020. A total of 100 wastewaters were collected from five governmental hospitals in Addis Ababa using a grap-sampling technique. All Enterobacteriaceae were screened for ESBLs production using cefotaxime and ceftazidime as per 29th CLSI guideline. Each screen positive for ESBLs production was confirmed by the combination disk method (CDT) and their antibiotic susceptibility pattern was done using the Kirby–Bauer disk diffusion method on Muller Hinton agar (MHA). Data were entered and summarized using SPSS version 20 software.

**Results:**

Of all Enterobacteriaceae, 48.3% were confirmed ESBLs-pE. The highest ratio of ESBLs-PE was observed in the adult ward (66.7%) and laundry unit effluent (58.8%). The highest ESBL producers were *E. coli* (21.8%) and *K. pneumoniae* (4.8%). The most elevated resistance level of ESBL producers were observed to cefotaxime (95.8%) and amoxicillin/clavunalate (93%). 64% of tested Enterobacteriaceae isolates were multi drug resistant (MDR).

**Conclusions:**

Higher magnitude of MDR and ESBLs-pE were present in the hospital wastewater. The majority of them were in the adult ward and laundry unit effluents. The most frequent ESBLs-pE was among *E.coli* and *K. pneumoniae*. Hence, Consistent infection prevention and control procedures should be in practice at each ward/unit.

## Introduction

Wastewater refers to water used by humans and loses its quality and usefulness that embrace waste liquid of domestic, agricultural, commercial sources, industries, and hospital sources. All these anthropogenic activities generates wastewater as a result of the physical, chemical, and biological quality of water deterioration [1]. The effluent from hospitals contains a lot of drug-resistant pathogens, a larger form of chemicals, solvents, disinfectants, and a lot of risky materials, such as pharmaceuticals and radionuclides than domestic sewerage [2]. As a result of these hospital effluent contains antibiotic residues that are enough to kill susceptible bacteria and at the same time increase the number of resistant bacteria [3]. The presence of the antibiotic-resistant microorganism in effluent and sewage system lines is a growing public health concern [4]. Since one of the main ways of diffusion of pathogenic and/or antibiotic-resistant microorganisms is thru water, soil, and air. This phenomenon results in multi-drug resistant (MDR) microorganisms that have been revealed in different water sources including rivers, lakes, groundwater, and drinking water [5–8]. The discharge of resistant bacteria to the receiving aquatic environment will create public health impact through, carrying the transmissible genes, by acting as a vector or reservoir of a resistant genes [9, 10].

The members of the Enterobacteriaceae are Gram-negative bacilli, which are usually resident in the gastrointestinal tract. An instance of such organism consists of *E. coli, K. pneumoniae, Enterobacter cloacae, Citrobacter freundii*, and *Proteus mirabilis.* In patients hospitalized in intensive care units (ICUs), the Enterobacteriaceae holds for about one-third of all cases of ICU-acquired pneumonia, one-third of all cases of ICU-acquired urinary tract infection, and 10–15% of ICU-acquired bloodstream infections [11].

Currently, the dominant antibiotics used for treating hospitalized patients infected by Gram-negative bacteria are the β-lactam antibiotics that inhibit transpeptidases taking part in bacterial cell wall synthesis. Sadly, these beta-lactam antibiotics will be deactivated by β-lactamase enzymes [12]. Persistent exposure of bacterial strains to a multitude of beta-lactam antibiotics has evoked a dynamic, continuous production and mutation of beta-lactamase in the bacteria resulting in the event of extended spectrum beta-lactamases (ESBLs) inflicting resistance to broad spectrum beta-lactam antibiotics [12–15].

ESBLs are beta-lactamases that confer resistance to the penicillins; first, second, and third-generation cephalosporins and monobactams by hydrolysis of these antimicrobials. In addition, these enzymes are pent-up by beta-lactamase inhibitors, such as clavulanic acid, sulbactam, and tazobactam. ESBLs are encoded on transferrable conjugative plasmids that facilitate widespread dissemination, not solely between the identical species of bacteria, but also across completely different species. Furthermore, these plasmids code for resistant to other classes of potent antimicrobial agents, insignificantly, aminoglycosides and fluoroquinolones [16]. Among the numerous ESBLs described in a variety of pathogens, CTX-M, TEM, and SHV types proved to be the most successful in terms of promiscuity and dissemination across various epidemiological niches [17].

The first ESBLs were isolated in the 1980s [18, 19]. ESBLs are isolated from a large type of Enterobacteriaceae (foremost in *E. coli and K. pneumoniae*), *Pseudomonas aeruginosa*, and *Capnocytophaga ochracea* [20]. The increasing prevalence of ESBL Enterobacteriaceae especially *K. pneumoniae* and *E. coli* worldwide is a source of explicit concern. The hospital sewage or the low effectiveness of hospital wastewater treatment plants may lead to the dissemination of ESBL-producing bacteria. The permanent presence of detectable antimicrobial levels within the hospital wastewater treatment may powerfully influence the environment, creating a selective pressure that would be ultimately responsible for the dominance of resistant microorganisms among those present in the habitat.

Our first objectives were to generate information on the occurrence of ESBLs producing Enterobacteriaceae (ESBLs-pE) and their antibiotic susceptibility pattern in Wastewater Released from the Governmental Hospital of Addis Ababa, Ethiopia. Since currently, almost all hospitals in Addis Ababa neither have wastewater treatment plants (WWTPs) nor functional waste stabilization bonds. Our second objective was to assess the frequency of the common Enterobacteriaceae and their antimicrobial susceptibility pattern.

## Methods

### Study setting

A cross-sectional study design was employed from April 1 to May 31, 2020, in Addis Ababa which is the Capital City of the Federal Democratic Republic of Ethiopia, the diplomatic capital of Africa, and the seat of different international and regional organizations. The city administration is divided into ten sub-city and 116 woredas. It hosts an estimated 3.2–38 million people, which is a 17% share of Ethiopia’s total urban population. Presently, Addis Ababa is experiencing an annual growth rate of 3.8% and is speculated to reach 4.7 million residents by 2030. The city covers a Landmass of 540 square kilometres. The city is located at the heart of the country, at an altitude ranging from 2100 m at Akaki in the south to 3000 m at Entoto Hill in the North [21].

Within the city, there are 13 government hospitals (five federal, six under Addis Ababa health bureau, one owned by police force, and one armed force hospital) distributed throughout 10 sub-cities. For this study, it was necessary to pick out major hospitals with a lot of instrumentation, a variety of medical services, higher beds and a large run of patients. Hence, hospitals having 200 or more beds and those which provide 10 or more types of medical services are considered as major. Seven governmental hospitals in Addis Ababa meet these criteria. Out of these, 5 hospitals are selected randomly to make a sample size of 38.5%. Ten percent or more samples is considered as a good sample size for small populations [22]. The selected hospitals included: Tikur Anbessa Specialized Hospital (TASH) having 543 beds and providing 26 different medical services, St. Paul’s Hospital Millennium Medical College (SPHMMC) having 337 beds and providing 25 different medical services, All African leprosy and tuberculosis rehabilitation training center (Alert hospital) having 241 beds and providing 16 different medical services, Yekatit 12 hospital medical college (Y12HMC) having 210 beds and providing 19 different medical services and Menelik ΙΙ referral hospital (MIIRH) having 203 beds and providing 15 different medical services [23].

Laboratory analysis was conducted at Ethiopian public health institute (EPHI) in clinical bacteriology and mycology national referral laboratory in collaboration with food microbiology laboratory. The EPHI laboratory has been accredited by Ethiopian National Accreditation Office.

### Sampling frequency and sampling technique

Hospitals that have a higher bed and serve many patients were selected in ten sub-city of Addis Ababa. Based on these five governmental hospitals were included in this study. In each hospital, six sampling sites are employed to collect hospital effluent. These are a manhole used to collect wastewater originating from an adult ward, pediatric ward, labor ward, laboratory unit, laundry unit, and a manhole of the septic tank (which hold effluent from all source and we term “Mixed” for this research purpose only). In addition, one sampling unit viz. MDR TB ward was incorporated to collect hospital effluent only in the Alert hospital.

A total of 100 discrete (that is 50 in the morning and 50 in the afternoon) wastewater samples were collected from each ward/unit wastewater collecting manholes at the sampling site with 4-h intervals in the study period. This encompassed discrete effluent of 24 from each Y12HMC and MIIRH, 20 from each SPHMMC and ALERT, and 12 discrete effluents from TASH.

A “Grab sampling technique” was applied to collect the most representative samples according to guidelines of wastewater sampling techniques stated on Environment Protection Authority (EPA) [24] and American Public Health Association (APHA) [25]. Discrete samples were collected in two rounds in each hospital for 2 months. The 1st and 2nd round samples were collected within 15-day intervals. In each round, the discrete samples were collected two times a day with 4-h intervals from each sampling site in each hospital in 150 ml cleaned and sterile microbiological glass bottles. Here 150 ml sterile glass container was used to collect 125 ml wastewater samples.

Hospitals’ effluent Samples were collected during their maximum activity period (usually 10:00 am- 2:30 pm) according to the method used by Nuñez and Moretton [10]. In addition, samples were collected near the center of the flow channel, at approximately 10–15 cm of the water depth, where the turbulence was at maximum and the possibility of settling was minimized. Grazing (skimming) the water surface or slogging the bottle was avoided. The first sample was collected at 10–10:30 AM, whereas the second sample was collected at 2–230 PM from each sampling site. After taking the sample, the neck of the bottle was wiped with 95% alcohol then the sample bottle labeled with the date, time of collection, and sampling site code number. All samples were collected manually and transported instantly to EPHI food microbiology and clinical laboratories with cold chin (4 °C) for bacteriological analysis within 6 h of collection. A pair of new, non-powdered, disposable gloves, a suitable gown, and an eye google was used each time while collecting samples to avoid personal contamination. In the same token, heavy-duty glove was used to clean and pick up the cover of manholes at the time of sample collection.

### Sampling site

In the present study, there were different wards and units in the selected hospitals. Each ward/unit generates wastewater which has different characteristics. Therefore, to locate in which sampling site the isolate was found, the hospital effluents were collected at different manholes of each unit/ward of the hospital. The hospital effluents were collected at the manhole of the adult ward, pediatric ward, delivery ward, laboratory unit, laundry service unit, and at sampling site named Mixed. Here mixed sample indicates a hospital effluent in which its origin held from different ward/units and flow together hence it was difficult to identify at which specific unit/ward it came. In addition, wastewater was collected from the MDR TB ward in the case of Alert hospital only. The sampling site manholes are located just at the outlet of wastewater of each ward/unit before being discharged into receiving water/ collecting septic tank and at the side of each ward/unit building. The geographical position of the sampling site/ unit was obtained and documented.

### Data collection procedure

The important information’s were recorded using a pre-developed data collection form by asking the authorized body (about sampling unit/site, wastewater disinfection, and disposal procedures), from record book/file (e.g., number of patients served during study period) and using google map application (for the geographical location of the sample). After the sampling unit/site at each hospital is identified, located, and its wastewater source recognized, then aseptically with care, the cover of each manhole is lifted to collect the wastewater. The name of the hospital and sampling unit, time and round of collection, as well as its geographical location, is recorded on pre-developed data collection format in addition to, labeling the collecting bottle at the time of sample collection. All of this information was collected by the principal investigator.

## Laboratory analysis

### Isolation and characterization of pure cultures

For isolation of the bacteria, a loopful of a well-mixed sample suspension was inoculated using the sterile inoculating loop onto MacConkey agar plate (Oxoid LTD, Basingstoke, Hampshire, England) and incubated aerobically at 37 °C for 18–24 h.

After incubation for 24 h. at 37 °C, bacterial colonies with distinct coloration and morphology were randomly picked up and sub-cultured onto another MacConkey agar plate for further purification. Then purified colonies with distinct presumptive colonies of each suspected bacterial species and fermentation on MacConkey agar are sub-cultured on tryptic soy agar (TSA)/nutrient agar (Oxoid LTD, Basingstoke, Hampshire, England) depending on the availability of media for the biochemical test.

For identification pure colony from non-selective nutrient agar/TSA was sub-cultured and identified based on the following biochemical tests: oxidase, indole, urea, motility, Lysine decarboxylase, citrate utilization, and triple sugar iron tests as per the standards of microbiology procedure including *E. coli* ATCC 25922, as a control culture [26]. Following purification and species identified, two–three purified colonies were preserved in skimmed milk at − 80 °C for further characterization, after each isolate was assigned a unique identification number.

### Screening isolates for ESBLs producing

Those Enterobacteriaceae that was resistant or reduced susceptibility to the screening indicator cephalosporin (cefotaxime and/or ceftazidime) was considered as suspicious of ESBLs production. In other words, isolates that showed an inhibition zone size of ≤ 27 mm for cefotaxime (30 μg) and/or ≤ 22 mm for ceftazidime (30 μg) were considered as suspicious ESBL producers and selected for confirmation for ESBLs production.

### Confirmation of ESBLs producing Enterobacteriaceae

Confirmation of suspicious ESBL-producing isolate was verified by the combination disk method (CDT) as delineated by the 29th edition CLSI guideline [27]. The test was performed using two cephalosporin antibiotics: ceftazidime (30 μg), and cefotaxime (30 μg) alone and in combination with beta-lactam inhibitor [ceftazidime- clavulanic acid (30/10 μg), and cefotaxime-clavulanic acid (30/10 μg)] by dispensing on 0.5 McFarland turbidity bacterial suspension inoculated Muller Hinton agar (MHA) plate (Oxoid LTD, Basingstoke, Hampshire, England) and then incubated overnight (18–24 h) at 37 °C as per 29th edition CLSI guideline. ESBL production was considered positive when ≥ 5 mm increase in the zone diameter for the ceftazidime or cefotaxime tested in combination with clavulanic acid vs. its zone when tested alone [27]. *E. coli* ATCC 25922 was used as a negative control throughout the tests as a non-ESBL culture.

### Antimicrobial susceptibility testing

Once the bacteria were isolated and identified from each sample collected, all Enterobacteriaceae isolates were assessed for a resistance pattern for 12 antibiotic agents using the Kirby-Bauer disk diffusion method on MHA in line with the 29th edition CLSI guideline [27]. Bacterial inoculums were prepared by suspending the freshly grown bacteria in 4–5 ml sterile normal saline and the turbidity was adjusted to that of a 0.5 McFarland standard. Then a prepared bacterial inoculums suspension (0.5 McFarland standards) was streaked on MHA using a sterile swap applicator stick and antimicrobial discs were placed.

The antibiotic discs used for this study were: trimethoprim/sulfamethoxazole (SXT, 1.25/3.75 μg), ciprofloxacin (CPR, 5 μg), tazobactam + piperacillin (TZP, 30 μg), cefoxitin (CXT, 30 μg), chloramphenicol (CHL, 30 μg), nitrofurantoin (F, 300 μg), amoxicillin/clavulanic acid (AMC, 20/10 μg), tobramycin (TOP, 10 μg), meropenem (MER, 10 μg), cefotaxime (CTX, 30 μg), cefepime (CFP, 30 μg), and ceftazidime (CAZ, 30 μg). The antibiotic discs used were the product of Abtek Biologicals Ltd, Liverpool, United Kingdom. Inhibition zones were measured using a ruler and isolates were categorized as resistant, intermediate, and susceptible for each antimicrobial agent using the breakpoint asset in line with 29th edition CLSI guidelines [27].

The isolates were going to be considered as MDR when they were resistant to three or more classes of antibiotics [28]. *E. coli* ATCC 25922 and *P. aeruginosa* ATCC 27853 were used for quality control throughout the antimicrobial susceptibility tests as recommended by 29th edition CLSI.

### Laboratory data quality assurance

Sample collection, handling, transportation, and microbiological analysis, and interpretation of results were carried out using standard operating procedures. Before the tangible procedure; reagents, antimicrobial disks, and media were checked for damage, storage problems, and expiry date. Laboratory equipment is appropriately cleaned and sterilized before use. Media was prepared according to the respective manufacturer’s instruction. Five percent of prepared media per batch was incubated overnight for sterility checkup. Quality control for the new batch was performed using ATCC 25922 *E. coli* standard control to cross-check the quality of antibiotics disks and culture media. For ESBLs confirmatory test *E. coli* ATCC 25922 (ESBLs negative) and *K. pneumoniae* ATCC 700603 (ESBLs positive), standard control strains are served at the time of the procedure [27].

### Data analysis and interpretation method

Data was entered and summarized using SPSS version 20 software (IBM Corporation, Armonk, NY, USA). Frequency and percentages of isolates, antibiotic susceptibility pattern of Enterobacteriaceae, and ESBLs-pE were calculated. Tables and figures have been employed for data demonstration.

### Statistical data quality assurance

Before data entry, data from the data collection form was cross-checked for its completeness and accuracy. Culture isolates and antibiotics susceptibility test results had been documented consciously ahead of entry to SPSS. Furthermore, data cleaning and double-data entry were implemented to assure the quality of the data.

## Results

### Distribution of gram-negative bacteria isolates against sampling unit and hospitals

A total of 100 hospital effluent samples were collected and analyzed for the presence of the Enterobacteriaceae family. Of these samples, 87% were tested positive and contained one or more than one type of isolates. Meanwhile, 183 non-duplicate Gram-negative bacteria were picked from MacConkey agar, 80.3% (147) belonging to Enterobacterial species. The remaining isolates included *Pseudomonas* spp. (2.2%), *Acinetobacter* spp. (4.4%), and other unidentified Gram-negative bacteria (13%).

Of 147 Enterobacteriaceae family isolates recovered, the highest distributions were from; laboratory unit 32 (21.8%) and mixed source wastewater 30 (20.4%). In this study, the highest number of isolated bacteria, irrespective of the total sample collected, were recovered from Y12HMC [34], while the least isolates were obtained from TASH [21] (Table 1).

**Table 1:** Distribution of Enterobacteriaceae isolate against within the hospital, sampling unit, time and round of sample collection, at selected governmental hospitals wastewater, Addis Ababa, Ethiopia from April 1 to May 31, 2020

Variables, *n* (%)		Enterobacteriaceae isolates *n* (%)									
		*E. coli*	*K. pneumonia*	*E. cloacae*	*Citrobacter *spp.	*K. ozanae*	*K. rhinoscler*	*K. oxytoca*	*M. morganii*	*Sallmonella *spp.	Other isolates*
Hospital	TASH, 21 (14.3)	6 (28.6)	2 (9.5)	4 (19)	1 (4.8)	3 (14.3)	3 (14.3)	1 (4.8)	0	1 (4.8)	0
	SPHMMC, 32 (21.8)	17 (53.1)	3 (9.4)	2 (6.3)	1 (3.1)	2 (6.3)	1 (3.1)	0	1 (3.1)	1 (3.1)	4 (12.5)
	ALERT, 28 (19)	12 (42.9)	3 (10.7)	2 (7.1)	4 (14.3)	0	0	2 (7.1)	2 (7.1)	1 (3.6)	2 (7.1)
	Y12HMC, 34 (23.1)	16 (47.1)	3 (8.8)	3 (8.8)	4 (11.8)	1 (2.9)	3 (8.8)	2 (5.9)	1 (2.9)	0	1 (2.9)
	MIIRH, 32 (21.8)	16 (50)	4 (12.5)	3 (9.4)	2 (6.3)	2 (6.3)	1 (3.1)	2 (6.3)	0	0	2 (6.3)
Sampling Unit	Adult ward, 21 (14.3)	13 (61.9)	1 (4.8)	2 (9.5)	4 (19)	0	0	0	0	1 (4.8)	0
	Pediatric ward, 19 (12.9)	9 (47.4)	2 (10.5)	2 (10.5)	1 (5.3)	2 (10.5)	1 (5.3)	1 (5.3)	1 (5.3)	0	0
	Laboratory unit, 32 (21.8)	15 (46.9)	4 (12.5)	3 (9.4)	1 (3.1)	1 (3.1)	4 (12.5)	1 (3.1)	1 (3.1)	0	2 (6.3)
	Laundry unit, 17 (11.6)	10 (58.8)	3 (17.6)	2 (11.8)	0	0	0	0	0	0	2 (11.8)
	Labor ward, 21 (14.3)	7 (33.3)	2 (9.5)	2 (9.5)	1 (4.8)	1 (4.8)	2 (9.5)	3 (14.3)	0	1 (4.8)	2 (9.5)
	Mixed source, 30 (20.4)	10 (33.3)	3 (10)	3 (1)	4 (13.3)	4 (13.3)	1 (3.3)	2 (6.7)	0	1 (3.3)	2 (6.7)
	MDR TB ward, 7 (4.8)	3 (42.9)	0	0	1 (14.3)	0	0	0	2 (28.6)	0	1 (14.3)
Time of collection	Morning, 71 (48.3)	33 (46.5)	10 (14.1)	7 (9.9)	4 (5.6)	1 (1.4)	4 (5.6)	3 (4.2)	3 (4.2)	3 (4.2)	3 (4.2)
	Afternoon, 76 (51.7)	34 (44.7)	5 (6.6)	7 (9.2)	8 (10.5)	7 (9.2)	4 (5.3)	4 (5.3)	1 (1.3)	0	6 (7.9)
Round of collection	First, 80 (54.4)	36 (45)	7 (8.8)	8 (10)	6 (7.5)	5 (6.3)	5 (6.3)	5 (6.3)	2 (2.5)	2 (2.5)	4 (5)
	Second, 67 (45.6)	31 (46.3)	8 (11.9)	6 (9)	6 (9)	3 (4.5)	3 (4.5)	2 (3)	2 (3%)	1 (1.5)	5 (7.5)
Total (*N* = 147)		67 (45.6)	15 (10.2)	14 (9.5)	12 (8.2)	8 (5.4)	8 (5.4)	7 (4.8)	4 (2.7)	3 (2)	9 (6.1)

*Other isolates were *Proteus* spp.*, Shigella* spp.*, Ent. aerogens, Edwardsella,* and *P. alkalifacience*

TASH = Tikur Ambesa Specialized Hospital, SPHHMMC = St. Paul’s Hospital Millennium Medical College, ALERT = All African leprosy and tuberculosis rehabilitation training center, Y12HMC = Yekatit 12 hospital medical college, MIIRH = Menilik ΙΙ referral hospital, MDR TB Ward = multidrug resistant tuberculosis ward

### Frequency of Enterobacteriaceae isolates

Among all Enterobacteriaceae, the most frequent isolates were *E. coli* (45.6%), *K. pneumoniae* (10.2%), and *E. cloacae* (9.5%), respectively. *E. coli* was predominantly isolated in laboratory effluent (22.4%; 15/67) followed by adult ward effluent (19.4%; 13/67). Meanwhile, *K. pneumoniae* was mostly obtained from laboratory effluent (26.7%; 4/15), laundry unit (20%; 3/15), and mixed source wastewater samples (20%; 3/15). *E. coli* and *K. pneumoniae* were the most frequent isolates identified within the pediatric ward (47.4%, 10.5%) and laboratory unit (46.9%, 12.5%), respectively, whereas *E. coli* and C*itrobacter* spp. were predominantly isolated Enterobacteriaceae within the adult ward, (61.9%, 19.5%) and mixed source effluent (33.3%, 13.3%), respectively (Table 1).

### Antimicrobial susceptibility pattern of Enterobacteriaceae

The prevalence of antimicrobial-resistant pattern for Enterobacteriaceae isolated ranged from 8.2 to 77.6% in wastewater isolates, with most of the strains susceptible to meropenem (MER) and nitrofurantoin (F). They showed high resistance to amoxicillin-clavulanic acid (77.6%) and trimethoprim/sulfamethoxazole (57.8%). In this study *E. coli, Citrobacter* spp., and *E. cloacae* revealed the highest resistance for amoxicillin-clavulanic acid of 85.1%, 75%, and 85.7%, respectively. However, K*. pneumoniae* showed the highest level of resistance to CTX (60%) (Table 2).

**Table 2:** Antimicrobial resistant pattern of Enterobacteriaceae identified from different sampling unit at selected governmental hospitals in Addis Ababa, Ethiopia from April 1 to May 31, 2020

Isolates (number)	Tested Antibiotics Number *n* (%)											
	SXT	CPR	TZP	CXT	CHL	F	AMC	TOB	MER	CTX	CFP	CAZ
*E. coli* (67)	43 (64.2)	34 (50.7)	9 (13.4)	14 (20.9)	4 (6)	0	57 (85.1)	18 (26.9)	3 (4.5)	35 (52.2)	32 (47.8)	29 (43.3)
*K. rhinoscler* (8)	4 (50)	4 (50)	0	2 (25)	3 (37.5)	0	7 (87.5)	4 (50)	0	4 (50)	4 (50)	4 (50)
*K. pneumonia* (15)	7 (46.7)	6 (40)	0	0	2 (13.3)	0	8 (53.3)	5 (33.3)	0	9 (60)	8 (53.3)	8 (53.3)
*Citrobacter* spp. (12)	8 (66.7)	7 (58.3)	1 (8.3)	7 (58.3)	5 (41.7)	2 (16.7)	9 (75)	2 (16.7)	2 (16.7)	6 (50)	5 (41.7)	6 (50)
*Sallmonella* spp. (3)	1 (33.3)	2 (66.7)	0	0	1 (33.3)	0	3 (100)	1 (33.3)	1 (33.3)	2 (66.7)	2 (66.7)	2 (66.7)
*E. cloacae* (14)	7 (50)	9 (64.3)	5 (35.7)	9 (64.3)	6 (42.9)	6 (42.9)	12 (85.7)	6 (42.9)	5 (35.7)	8 (57.1)	8 (57.1)	8 (57.1)
*K. oxytoca* (7)	2 (28.6)	3 (42.9)	0	1 (14.3)	1 (14.3)	0	3 (42.9)	2 (28.6)	0	3 (42.9)	2 (28.6)	3 (42.9)
*K. ozanae* (8)	5 (62.5)	2 (25)	1 (12.5)	4 (50)	2 (25)	0	5 (62.5)	3 (37.5)	0	5 (62.5)	5 (62.5)	4 (50)
*M. morganii* (4)	3 (75)	3 (75)	0	0	0	2 (50)	3 (75)	0	0	3 (75)	3 (75)	3 (75)
Other isolates (*9)*	5 (55.6)	7 (77.8)	3 (33.3)	2 (22.2)	2 (22.2)	2 (22.2)	7 (77.8)	2 (22.2)	1 (11.1)	4 (44.4)	4 (44.4)	4 (44.4)
Total resistance (*N* = 147)	**85 (57.8)**	**77 (52.4)**	**19 (12.9)**	**39 (26.5)**	**26 (17.7)**	**12 (8.2)**	**114 (77.6)**	**43 (29.3)**	**12 (8.2)**	**79 (53.7)**	**73 (49.7)**	**71 (48.3)**

Note: SXT = Sulfamethoxazole-trimethoprim, CRP = Ciprofloxacin, TZP = Tazobactam/Piperacillin, CXT = Cefoxitin, CHL = Chloramphenicol, F = nitrofurantoin, AMC = Amoxicillin-Calvulanic acid, TOB = Tobramycine, MER = Meropenem, CTX = Cefotaxime, CFP = Cefepime, CAZ = Ceftazidime. *Other isolates were *Proteus* spp., *Shigella* spp., *Ent. aerogens, Edwardsella,* and *P. alkalifacience*

Out of 147 Enterobacteriaceae strains tested, 125 (85%) were found resistant to at least one or more antibiotics tested. Hence, 64% (94/147) of tested Enterobacteriaceae were MDR strain. A total of 62.7% *E. coli*, 83.3% *Citrobacter* spp., and 64.3% *E. cloacae* were identified as the predominant MDR isolates within species (Table 3).

**Table 3:** Level of antibiotic resistant of Enterobacteriaceae identified from different sampling units of selected governmental hospitals, Addis Ababa, Ethiopia from April 1 to May 31, 2020

Isolates (number)	Level of antibiotics resistant *n* (%)								Total MDR-E (≥ R3)
	R0	R1	R2	R3	R4	R5	R6	R7	
*E. coli* (67)	6 (9)	8 (11.9)	11 (16.4)	8 (11.9)	4 (6)	7 (10.4)	8 (11.9)	5 (7.5)	42 (62.7)
*K. rhinoscler* (8)	1 (12.5)	0	1 (12.5)	2 (25)	0	0	1 (12.5)	2 (25)	5 (62.5)
*K. pneumonia* (15)	6 (40)	1 (6.7)	0	0	0	1 (6.7)	2 (13.3)	5 (33.3)	8 (53.3)
*Citrobacter* spp. (12)	0	0	2 (16.7)	2 (16.7)	2 (16.7)	2 (16.7)	1 (8.3)	1 (8.3)	10 (83.3)
*Sallmonella* spp. (3)	0	1 (33.3)	0	0	0	0	0	2 (66.7)	2 (66.7)
*E. cloacae* (14)	2 (14.3)	0	3 (21.4)	1 (7.1)	0	0	1 (7.1)	1 (7.1)	9 (64.3)
*K. oxytoca* (7)	4 (57.1)	0	0	0	0	1 (14.3)	0	1 (14.3)	3 (42.9)
*K. ozanae* (8)	2 (25)	0	1 (12.5)	0	0	1 (12.5)	2 (25)	0	5 (62.5)
*M. morganii* (*4)*	1 (25)	0	0	0	0	0	1 (25)	2 (50)	3 (75)
Other isolates (9)	0	0	3 (33.3)	1 (11.1)	1 (11.1)	2 (22.2)	0	0	6 (66.7)
Total (N = 147)	22 (15)	10 (6.8)	21 (14.3)	14 (9.5)	7 (4.8)	14 (9.5)	16 (10.9)	19 (12.9)	94 (64)

R0: resistant to no antibiotics, R1–7: resistant to 1, 2, 3, 4, 5, 6, 17 antibiotics and ≥ R3 stands for resistance to 3 or more antibiotics from different classes,MDR-E stands for multi-drug resistant Enterobacteriaceae *Other isolates were *Proteus* spp., *Shigella* spp., *Ent. aerogens, Edwardsella,* and *P. alkalifacience*

### Magnitude of ESBLs producing Enterobacteriaceae

**Fig. 1:**
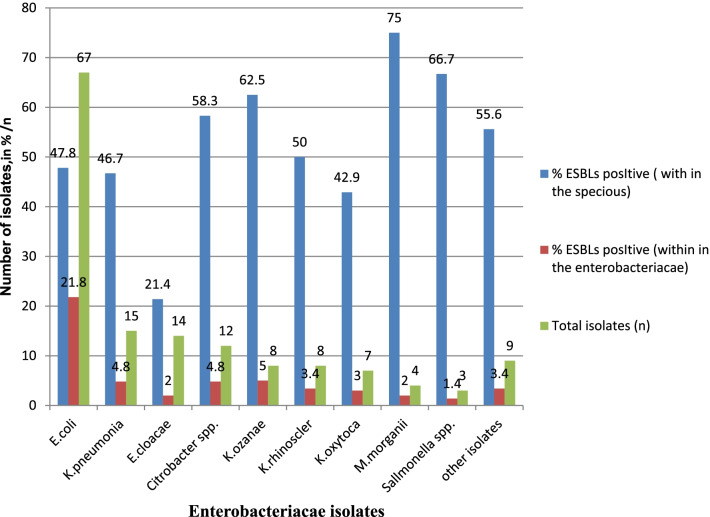
Frequency of the ESBLs producing Enterobacteriaceae within the species and the total Enterobacteriaceae from different sampling units at selected governmental hospitals wastewater, in Addis Ababa, Ethiopia from April 1 to May 31, 2020

Of all Enterobacteriaceae (*N* = 147) 81isolates were suspected as potential ESBLs producing with screening method of cefotaxime zone of inhibition ≤ 27 mm and ceftazidime zone of inhibition ≤ 22 mm. Out of 81 ESBLs potential Enterobacteriaceae*,* 71 (87.7%) were found to be ESBLs producing isolates by the combination disk test. The overall magnitude of ESBLs producing Enterobacteriaceae (ESBLs-pE) was 48.3% (71/147) (Table 4), with the highest percentage found in *E. coli*, (21.8%; 32/147), *K. pneumoniae* (4.8%; 7/147), and *Citrobacter* spp. (4.8%; 7/147), while the lowest ratio observed in *Salmonella* spp. (1.4%; 2/147), respectively. The occurrence of ESBLs producers differs strongly within different species of Enterobacteriaceae. The highest within-species frequency of ESBLs production was recovered among *M. morganii* (75%) pursued by *Salmonella* spp. (66.7%) and *K. ozaenae* (62.5%), respectively. However, least within-species ESBLs production was found in *E. cloaca* (21%) (Fig. 1).

**Table 4:** Distritution of MDR and ESBLs producing Enterobacteriaceae within the sampling units, the hospital, time and round of wastewater collection at selected governmental hospitals, in Addis Ababa, Ethiopia from April 1 to May 31, 2020

Variables (isolates, *n*)		MDR Enterobacteriaceae *n* (%)	Potential ESBLs producing Enterobacteriaceae		Non-ESBL potential Enterobacteriaceae *n* (%)
			ESBLs test result *n* (%)		
			Positive	Negative	
Wastewater Sampling unit	Adult ward (21)	15 (71.4)	14 (66.7)	1 (4.8)	6 (28.6)
	Paediatric ward (19)	10 (52.6)	7 (36.8)	2 (10.5)	10 (52.6)
	Laboratory unit (32)	20 (62.5)	13 (40.6)	2 (6.3)	17 (53.1)
	Laundry unit (17)	12 (70.6)	10 (58.8)	2 (11.8)	5 (29.4)
	Labor ward (21)	13 (61.9)	10 (47.6)	2 (9.5)	9 (42.9)
	Mixed source (30)	18 (60)	13 (43.3)	13 (43.3)	16 (53.3)
	MDR TB ward (7)	6 (85.7)	4 (57.1)	1 (14.3)	3 (42.9)
Wastewater collected Hospitals	TASH (21)	16 (76.2)	11 (52.4)	4 (19)	6 (28.6)
	SPHMMC (32)	22 (68.8)	15 (46.9)	3 (9.4)	14 (43.8)
	ALERT (28)	20 (71.4)	19 (67.9)	0	9 (32.1)
	Y12HMC (34)	19 (55.9)	16 (47.1)	2 (5.9)	16 (47.1)
	MIIRH (32)	17 (53.1)	10 (31.3)	1 (3.1)	21 (65.6)
Wastewater collected Time	Morning (71)	45 (63.4)	34 (47.9)	5 (7)	32 (45.1)
	Afternoon (76)	49 (64.5)	37 (48.7)	5 (6.6)	34 (44.7)
Wastewater collected round	First (80)	52 (65)	39 (48.8)	9 (11.3)	32 (40)
	Second (67)	42 (62.7)	32 (47.8)	1 (1.5)	34 (50.7)
Total *N* = 147		94 (64)	71 (48.3)	10 (6.8)	66 (44.9)

TASH = Tikur Ambesa Specialized Hospital, SPHHMMC = St. Paul’s Hospital Millennium Medical College, ALERT = All African leprosy and tuberculosis rehabilitation training center, Y12HMC = Yekatit 12 hospital medical college, MIIRH = Menilik ΙΙ referral hospital, MDR TB Ward = multidrug resistant tuberculosis ward

### Distribution of MDR and ESBLs producing Enterobacteriaceae

The distribution of MDR, ESBLs producing and non-ESBL potential Enterobacteriaceae against the variables are presented in Table 4 below. From all hospital wastewater collected for this study purpose, the highest ratio of ESBLs-pE within the sampling unit was observed in the adult ward effluent (66.7%) followed by the laundry unit (58.8%) and labor ward (47.6%), respectively, whereas the least proportion was recovered in the pediatric ward effluent (36.9%). Similarly with less difference; the elevated MDR isolates within the sampling unit were identified in the adult ward effluent (71.4%) pursued by the laundry unit (70.6%) and laboratory unit (62.5%) correspondingly, while the lowest ratio was found, in the pediatric ward (52.6%). In MDR TB ward wastewater which was collected only in ALERT hospital, the proportions of ESBLs-producing and MDR Enterobacteriaceae within the sampling unit were 57.1% and 85.7%, respectively.

The magnitude of ESBLs producing and MDR Enterobacteriaceae in the wastewater were different in the five hospitals. The highest occurrence of ESBLs-pE within the hospital according to the CDT identification method was found in wastewater from the ALERT (67.9%), followed by TASH (52.4%) and Y12HMC (47.1%), respectively. Whereas the least ESBLs-pE within the hospital occurred in MIIRH (31.2%).On the contrary, the elevated MDR isolates within the hospital were observed in wastewater of TASH (76.2%) and ALERT (71.4%), while the lowest ratio was found in the same way as ESBL producer isolates in MIIRH (53.1%). Furthermore, the distribution of MDR and ESBLs producing Enterobacteriaceae within the sampling units, time of collection and round of wastewater collection at five governmental hospitals is presented in Table 5. The occurrence of ESBLs was not statistically significant among Enterobacteriaceae isolated from all independent variables (*p* > 0.05).

**Table 5:** Occurrence of MDR and ESBLs producing Enterobacteriaceae within the hospitals sampling units, time and round of wastewater collection at selected governmental hospitals, in Addis Ababa, Ethiopia from April 1 to May 31, 2020

Hospitals isolates *n*, vs. ESBLs positive and MDR yes, *n* (%)			Sampling unit *n* (%)							Wastewater collected time		Wastewater collected round	
			Adult ward	Pediatric ward	Laboratory	Laundry	Labor ward	Mixed source	MDR TB ward	Morning	Afternoon	First	Second
Sample collected Hospitals	TASH,21	ESBLs positive 11 (52.4)	1 (9.1)	2 (18.2)	2 (18.2)	1 (9.1)	2 (18.2)	3 (27.3)	NC	4 (36.4)	7 (63.6)	11 (100)	NC
		MDR yes 16 (31.2)	1 (6.3)	4 (25)	4 (25)	1 (6.3)	3 (18.8)	3 (18.8)	NC	8 (50)	8 (50)	16 (100)	NC
	SPHMMC,32	ESBLs positive 15 (46.9)	2 (13.3)	3 (20)	2 (13.3)	4 (26.7)	NC	4 (26.7)	NC	7 (46.7)	8 (53.3)	4 (26.7)	11 (73.3)
		MDR yes 22 (68.8)	4 (18.2)	3 (13.6)	4 (18.2)	5 (22.7)	NC	6 (27.3)	NC	11 (50)	11 (50)	9 (40.9)	13 (59.1)
	ALERT,28	ESBLs positive 19 (67.9)	4 (21.1)	NC	6 (31.6)	1 (5.3)	4 (21.1)	NC	4 (21.1)	9 (47.7)	10 (52.6)	12 (63.2)	7 (36.8)
		MDR yes 20 (71.4)	3 (15)	NC	6 (30)	1 (5)	4 (20)	NC	6 (30)	10 (50)	10 (50)	11 (55)	9 (45)
	Y12HMC,34	ESBLs positive 16 (47.1)	4 (25)	2 (12.5)	1 (6.3)	0	3 (18.8)	6 (37.5)	NC	8 (50)	8 (50)	9 (56.3)	7 (43.8)
		MDR yes 19 (55.9)	4 (21.1)	3 (15.8)	1 (5.3)	0	4 (21.1)	7 (36.8)	NC	8 (42.1)	11 (57.9)	9 (47.4)	10 (52.6)
	MIIRH,32	ESBLs positive 10 (31.2)	3 (30)	0	2 (20)	4 (40)	1 (10)	0	NC	6 (60)	4 (40)	3 (30)	7 (70)
		MDR yes 17 (53.1)	3 (17.6)	0	5 (29.4)	5 (29.4)	2 (11.8)	2 (11.8)	NC	8 (47.1)	9 (52.9)	7 (41.2)	10 (58.8)
Total, *N* = 147		ESBLs positive *n* = 71 (48.3%)	14 (19.7)	7 (9.9)	13 (18.3)	10 (14.1)	10 (14.1)	13 (18.3)	4 (5.6)	34 (47.9)	37 (52.1)	39 (54.9)	32 (45.1)
		MDR yes *n* = 94 (64%)	15 (16)	10 (10.6)	20 (21.3)	12 (12.8)	13 (13.8)	18 (19.1)	6 (6.4)	45 (47.9)	49 (52.1)	52 (53.3)	42 (44.7)

TASH = Tikur Ambesa Specialized Hospital, SPHHMMC = St. Paul’s Hospital Millennium Medical College, ALERT = All African leprosy and tuberculosis rehabilitation training center, Y12HMC = Yekatit 12 hospital medical college, MIIRH = Menilik ΙΙ referral hospital, MDR TB Ward = multidrug resistant tuberculosis ward, NC = sample not collected

In multivariate analysis using logistic regression, occurrences of ESBLs-pE in wastewater haven’t statistically significant association with hospitals, sampling units, round and time of wastewater collection

### Antibiotics susceptibility pattern of ESBLs producing Enterobacteriaceae

**Fig. 2:**
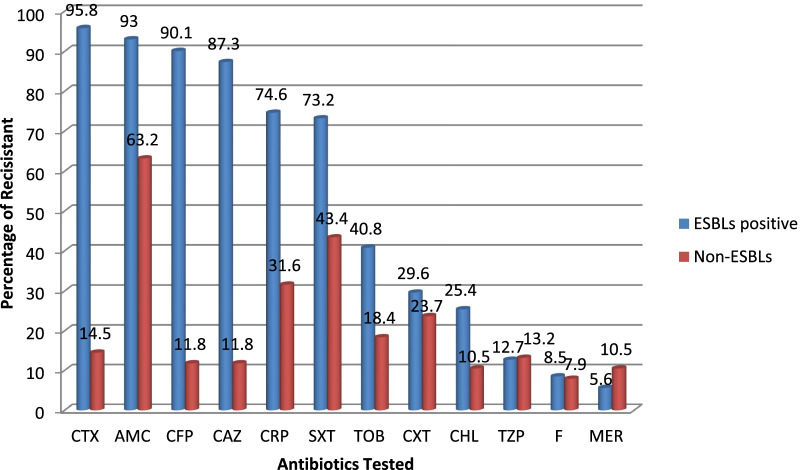
Antibiotics resistant pattern of ESBLs positive and Non-ESBLs Enterobacteriaceae to different classes of antibiotics at selected governmental hospitals, Addis Ababa, Ethiopia, from April 1 to May 31, 2020. SXT = Sulfamethoxazole-trimethoprim, CRP = Ciprofloxacin, TZP = Tazobactam/Piperacillin, CXT = Cefoxitin, CHL = Chloramphenicol, F = nitrofurantoin, AMC = Amoxicillin-Clavulanic acid, TOB = Tobramycine, MER = Meropenem, CTX = Cefotaxime, CFP = Cefepime, CAZ = Ceftazidime

The antibiotic-resistant profile for ESBLs producing and non ESBLs producer Enterobacteriaceae are displayed in Fig. 2. The antibiotics susceptibility pattern of ESBLs-pE was also performed to potential active antibiotics, such as carbapenems (meropenem), quinolone/fluoroquinolone (ciprofloxacin), cephamycine (cefoxitin), and aminoglycoside (tobramycin) drug categories. In addition, it was tested for combination drugs (amoxaciline-clavunalate and piperacillin-tazobactam), phenicol (chloramphenicol), nitrofuran (nitrofurantoin), and folate pathway antagonist (trimethoprim-sulfamethoxazole) drug families.

The predominant ESBLs-pE was found to be more than 85% resistant, the highest resistant levels were observed on cefotaxime (95.8%), amoxacilline/clavunalate (93%), and cefepime (90.1%). The most common co-resistance rates among the ESBLs-pE isolates to ciprofloxacin and trimethoprim-sulfamethoxazole were 74.6% and 73.2%, respectively. Meanwhile, aminoglycoside and cephamycine such as tobramycine (40.8%) and cefoxitine (29.6%) showed reduced efficacy against the ESBLs-pE*.* However, the most active drugs for ESBLs producing isolates were meropenem, nitrofurantoin, and piperacillin/tazobactam with susceptibility 94.4%, 88.7%, and 87.3% correspondingly.

Non-ESBLs producers Enterobacteriaceae showed a resistant level of 63.2%, 43.4%, and 31.6% to amoxacilline/clavunalate, trimethoprim-sulfamethoxazole, and ciprofloxacin, respectively. In addition, nitrofurantoin, chloramphenicol, and meropenem showed the least resistant with 7.9%, 10.5 and 10.5%, respectively.

**Fig. 3:**
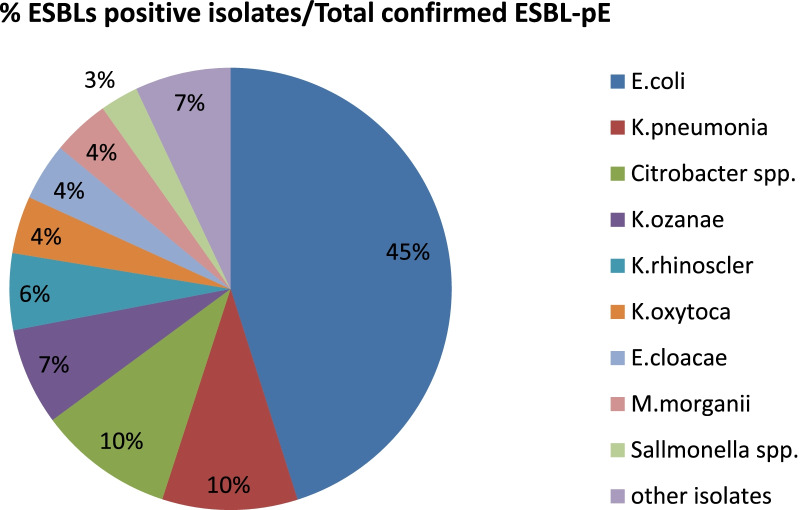
Percentage of ESBLs positive isolates within the total confirmed ESBLs producing Enterobacteriaceae at selected governmental hospitals wastewater, Addis Ababa, Ethiopia, from April 1 to May 31, 2020

Out of confirmed ESBLs-pE, the highest ESBLs production was observed among *E. coli* with 45% (32/71) prevalence. While the least ESBLs production from the total ESBLs positive Enterobacteriaceae was detected in *salmonella* spp. with 2.8% (2/71) ratio (Fig. 3).

## Discussion

The occurrence of antibiotic-resistant and ESBLs-pE from hospital wastewater could be exceptionally problematic because of the ability of nosocomial pathogens to transfer antibiotic resistance genes among different hosts and environments [29]. The dissemination of MDR bacteria via hospital wastewater is a usable cause for concern [30], because it is reasonable that MDR bacteria are selected mostly in hospitals and taken away by wastewater. This study, explains the prevalence, the occurrence of MDR, and ESBLs-pE from the five selected governmental hospitals wastewater in Addis Ababa, Ethiopia.

### Prevalence of Enterobacteriaceae isolates

In the present study, the most frequent Enterobacteriaceae isolates were *E. coli* (45.6 %), *K. pneumoniae* (10.2 %), and *E. cloacae* (9.5%). Our findings were comparable figure to other studies, in Addis Ababa; *E. coli* (32%), *K. pneumonia* (15%) and *E. cloacae* (6%) [31], in South Eastern, Nigeria; *E.coli* (26.2%) [32], in Luzhou City in Sichuan province, China; *E. coli* (56.5%), *K. pneumoniae* (27.4%) and *Enterobacter* spp. (8.1%) [33], in Bangladesh*; E. coli* and *Klebsiella* spp. (30.7% each) and *Enterobacter* spp. (25%) [34]. However, our finding was a little dissimilar to other studies conducted, in Northwest Ethiopia; from hospital environment *Klebsiella* spp. (29.2%), *E. coli* (12.3%) and *Enterobacter* spp. (3.1%) [35], in Mekelle: from untreated hospital wastewater *Klebsiella* spp. (25.9%), and *E. coli* (21.2%) [36], in Biratnagar, Nepal; from effluents of different hospitals sewage *E .coli* (34.7%), *Citrobacter* (21.7%), *Enterobacte*r (21.7%), and *Klebsiella* (13%) [37]. These variations might be due to sample type (inanimate object and swage of the hospital), study period, sample size, and type of pathogen infecting patients at the time of sample collection.

### Antibiotics resistant pattern of Enterobacteriaceae isolates

In the present study, the overall prevalence of antimicrobial-resistant pattern for Enterobacteriaceae isolated ranged from 8.2 to 77.6% in wastewater isolates, with most of the strains susceptible to meropenem (MER) and nitrofurantoin (F). This finding was in line with a study conducted in Rio de Janeiro, Brazil, with a 0–83% resistance range for Gram-negative isolates, and most strains susceptible to meropenem [38].

In this study, out of 147 Enterobacteriaceae strains tested, 125 (85%) were found resistant to at least one or more antibiotics tested. Meanwhile, Enterobacteriaceae strains showed the highest resistance to amoxicillin-clavulanic acid (77.6%) followed by trimethoprim/sulfamethoxazole (57.8%), cefotaxime (53.7%), and ciprofloxacin (52.4%).This finding more or less correlates with other studies conducted in China; trimethoprim/sulfamethoxazole (77.4%), amoxicillin-clavulanic acid (66.1%), and ciprofloxacin (61.3%) [33] had relatively higher resistance. However, our finding disagrees with the previous study conducted, where lower resistant proportion reported, in Northwest Ethiopia (Gondar): trimethoprim/sulfamethoxazole (29.8%), cefotaxime (23.8%), ciprofloxacin (10.6%) [35], and in Bangladesh; ciprofloxacin (23%) [34].

A study done in China, showed high resistance of Enterobacteriaceae for cefotaxime (100%), meropenem (51.6%), and chloramphenicol (48.4%) [33], contradicting the results presented herein, where less resistance was observed for cefotaxime (53.7%), chloramphenicol (17.7%) and meropenem (8.2%), again in Rio de Janeiro, Brazil: from the influent wastewater Gram-negative isolates showed resistance against cefotaxime (44%), trimethoprim/sulfamethoxazole (34%), ciprofloxacin (17%), and meropenem (3%) [38], which slightly deviated from current study except for meropenem.

In the present study, MDR strains were mostly observed in the tested Enterobacteriaceae isolates by 64%. Almost similar MDR isolate results with ours were recorded in a study carried out, in Northwest Ethiopia (Gondar) 81.5% (from hospital environment) [35], in Mekelle: 61.5% (from untreated hospital wastewater) [36], in Biratnagar, Nepal; 69.6% [37], in China: 85.5% [33]. However, our report contradicted the previous study conducted in South Eastern, Nigeria (from three hospital effluents), where all the Enterobacteriaceae isolates recovered (*E. coli* and *Salmonella* spp.) were MDR although their patterns of resistance varied [32]. In the same talk in this study, a total of 83.3% *Citrobacter* spp.*,* 64.3 % *E. cloacae*, 62.7% *E. coli*, and 53.3% *K. pneumoniae* isolates were identified as the predominant MDR. Our finding was concordant with other previous studies, where the common MDR isolates were, in Addis Ababa; *Citrobacter* (100%), *E. cloacae* (66.7%), and *E. coli* (28.6%) [31], in Biratnagar, Nepal; *Enterobacter spp.* (100%), *Citrobacter* spp. (80%), *E. coli* (62.5%), *Klebsiella* spp. (33.3%) [37]. However, our finding dissimilar with a study carried out in China [*E. coli* (91.4%) and *K. pneumoniae* (94.1%)] [33], Ibadan, Nigeria [*E. coli* (94.8%)] [39], and in Biratnagar, Nepal [*Enterobacter* spp. (100%)] [37], where the highest MDR proportion for *E. coli*, *K. pneumonia*, and *Enterobacter* spp. were indicated.

### Magnitude of extended spectrum B-lactamase producing Enterobacteriaceae

In the present study, of all Enterobacteriaceae 55.1% were suspected as potential ESBLs producing, and 87.7% of them were confirmed ESBLs producing isolates. A little comparable result was reported in Dubai, UAE by Khan et al. 2020: among all isolates from municipality wastewater 57.4% suspicious and 25.7% confirmed ESBLs producer Enterobacteriaceae were reported [40], in Northern Italy: 45.4% beta-lactamases producing Enterobacteriaceae were recovered from WWTPs [41]. The difference with our result might be due to the type of sample used (hospital vs. municipality wastewater), the method used to confirm potential ESBLs producer (CDT vs. DDST), and sample size.

According to the present study, the overall magnitude of ESBLs-pE was 48.3% which is almost in line with a study conducted in Rio de Janeiro, Brazil [38], and Nepal [37] with ESBLs producer isolates of 39%, and 30.4%, respectively. In contrast to the current study, other studies conducted in Ethiopia and other countries reported a lower prevalence of ESBLs-pE, in Addis Ababa: 25% from hospital wastewater [31], in Northwest, Ethiopia: 14.8% from hospital environment [42], and in Austria: 27.4% from activated sludge [43], were recovered [43]. The difference in the prevalence of ESBLs producers in different studies from wastewater isolates might be due to differences in geographic areas, source of sample, period of study (ESBL rapidly changing over time), sample size, method of ESBL detection, and an infection control system.

In the current study, the highest percentage of ESBLs-pE was detected in *E. coli* (45.1%), *K. pneumoniae* (9.9%), and *Citrobacter* spp. (9.9%) that is comparable results to a study conducted in, Austria with *E. coli* (65.6%), and *K. pneumoniae* (22.6%) [43] and in Ibadan, Nigeria *E.coli* (29.3%) [39]. However, the highest ESBLs producer prevalence was documented in *K. pneumoniae* than *E. coli* or other Enterobacteriaceae spp. in other studies which contradict the present study. Hence, the predominant ESBLs-pE isolate in other studies were, in Addis Ababa; *Citrobacter* spp. (33.3%), *K. pneumonia* (33.3%), and *E. coli* (20%) [31], in Northwest, Ethiopia, *K. pneumoniae* (42.10%), and *E. coli* (35.09%) [42], in Rio de Janeiro, Brazil: *K. pneumonia* (41.5%), and *E. coli* (12.2%) [38], and in Nepal; *Enterobacter* spp. (60%), Citrobacter spp. (40%) and *E. coli* (25%) [37]. This variation is occurred because of the difference in, wastewater type (hospital vs. municipality), source of wastewater contaminant, the prevalence of microbes, geographical location, and disease epidemiology.

The occurrence of ESBLs producers differs strongly within different species of Enterobacteriaceae*.* The highest within-species frequency of ESBLs production was recovered among *M. morganii* (75%) pursued by *Salmonella* spp. (66.7%) and *K. ozaenae* (62.5%), respectively. Meanwhile, least within-species ESBLs production was found in *E. cloaca* (21%). The difference of the occurrence of ESBLs producer between within ESBLs producer and species might be the variation of, the number of isolates recovered from the sample, and the isolate compared with (that is comparison within among ESBLs producer Enterobacteriaceae vs. within among each species).

### Distribution of MDR and ESBLS producing Enterobacteriaceae

In the present study, of all MDR Enterobacteriaceae, 73.4% were ESBLs producer, whereas only 26.6% of them were non-ESBLs producer Enterobacteriaceae. The magnitude of ESBLs producing and MDR Enterobacteriaceae in the wastewater were different in the five hospitals. The highest occurrence of ESBLs-pE within the hospital according to CDT identification method were found in wastewater from the ALERT (67.9%), followed by TASH (52.4%) hospitals, whereas the least ESBLs-pE were detected in the MIIRH hospital (31.2%). In contrary; the elevated MDR isolates within hospital were observed in wastewater of TASH (76.2%) and ALERT (71.4%), while the lowest ratio was found MIIRH (53.1%). Like our study report, there were different ESBLs producer occurrence within country hospital effluent, in Ibadan, Nigeria: more ESBLs producer was found in a privately-owned hospital (33.3%) than a State Government-owned hospital (29.1%) [39], in Europe: the elevated ESBLs-pE was found in effluents from the Slovenian general hospital, followed by the Austrian private rehabilitation clinic and the Austrian private surgery clinic [44].

From all hospital wastewater collected for this study purpose, the highest ratio of ESBLs-pE within sampling unit was observed in adult ward effluent (66.7%) followed by laundry unit (58.8%), whereas the least proportion was recovered in pediatric ward (36.9%). Meanwhile, the elevated MDR isolates within sampling unit were identified in adult ward effluent (71.4%) pursued by laundry unit (70.6%), whereas they were the lowest ratio in pediatric ward effluent (52.6%).The majority of the preceding publication on antibiotic resistant profile of pathogenic microbes has been focused towards crude hospital wastewater rather than at each refined source of it. As a result, it was difficult to compare our result directly with other studies conducted from hospital wastewater, for example a study conducted in the hospital environment in Gondar, reported from an inanimate object of medical ward, surgical ward and Gyn-obs ward ESBLs-pE of 52.6%, 10.5% and 5.3%, respectively [42].

In this study, the magnitude of MDR and ESBLs-pE obtained from all wastewater samples were higher in the afternoon (64.5% and 52.1%) than in the morning wastewater collected (59.2% and 47.9%). This difference may be happen (even if not statistically significant, *p* > 0.05) because of the majority of medical activity performed at the afternoon time and outpatients number increasing at the afternoon due to transportation and other reasons.

### Antibiotics susceptibility pattern of ESBLs producing Enterobacteriaceae

In current study, the predominant ESBLs-pE were found to be more than 85% resistant to the antibiotic, such as cefotaxime (95.8%), AMC (93%), cefepime (90.1%), and ceftazidime (87.3%). These were in close agreement with other study done in, Northwest Ethiopia; amoxicillin/clavulanic acid (100%) and ceftazidime (100%), Dubai, UAE; from municipality wastewater, cefotaxime (86%) and ceftazidime (77%) [40], Austria; amoxicillin/clavulanic acid (53.1%) [43] had higher resistance for ESBLs-pE.

In this study, the most common higher co-resistant rates among the ESBLs-pE isolates were 74.6% for ciprofloxacin and 73.2% for SXT. Aminoglycoside and cephamycine antibiotic groups such as tobramycine (40.8%) and cefoxitine (29.6%) show reduced efficacy to the ESBLs-pE. Our result was comparable with other studies taken place, in Northwest Ethiopia; ciprofloxacin (43.9%), and trimethoprim/sulfamethoxazole (SXT) (64.9%) [42], in Austria; ciprofloxacin (56.3%), trimethoprim/sulfamethoxazole (50%) and cefoxitin (25%) [43].

In our work, the most active drugs for ESBLs producing isolates observed are meropenem, nitrofurantoin, and piperacillin/tazobactam with susceptibility 94.4%, 88.7%, and 87.3%, respectively. The findings of this study concordance with prior reports conducted in Austria; meropenem (100%), and piperacillin/tazobactam (90.6%) had good susceptibility level [43].

Non-ESBLs producer*s* Enterobacteriaceae were 63.2%, 43.4%, 31.6% and 23.7% resistant to amoxicillin/clavulanic acid, trimethoprim/sulfamethoxazole, ciprofloxacin and Cefoxitin, respectively. However, nitrofurantoin, chloramphenicol and meropenem showed the least resistant with 7.9%, 10.5 and 10.5%, respectively. We observed in the present study ESBLs producer isolates were more resistant to the tested antibiotics than non-ESBLs producer Enterobacteriaceae*.* This difference might be from the resistant gene on ESBLs producer, which also contributed the isolate to develop resistance to other antibiotics too.

### Strength of the study

Our study tried to collect the wastewater at their utmost source, which enables to take preventive measures and at large sample size (in relative to the previous study) to be representative. This study conducted at different governmental higher hospitals to display the extent of distribution of MDR and ESBLs-pE in each hospital sampling units/sites.

### Limitation of the study

In this study only wastewater was used, hence it was unable to differentiate the source of resistant bacteria-either it was from clinical isolates or the sewage system.

Our study was unable to select all antimicrobial agents commonly used for resistance evaluation due to the fact that some of them were not available during the period of study.

The major limitation of the study was that ESBL detection was only performed phenotypically using CDT method, it was better to include a genotypic method of detection.

Some sources of hospital wastewater were not incorporated in the study. Therefore, to generalize the distribution of MDR and ESBLs-pE in hospital wastewater, it was better to assess all sources of wastewater in selected hospitals.

It would have a better figurative data if the study also included private hospitals, wastewater found in Addis Ababa and WWTP of the city.

## Conclusions

The hospital wastewater released directly into urban sewerage systems without appropriate disinfection or treatment is a serious environmental problem of the day. In this study, there was high magnitude of MDR & ESBLs-pE (≈ 65% vs. 50%) from the wastewater of selected governmental hospitals in Addis Ababa, Ethiopia. The majority of them were in the adult ward and the laundry unit effluents. In addition, the most frequent ESBLs-pE were *E. coli*, *K. pneumoniae* and *Citrobacter* spp. In addition, ESBLs-pE showed high rate of resistance to all tested antibiotics as compared to non-ESBLs-E. This is a warning threat to such infection via contamination of food and water from rivers and urbane drainage which will be polluted through untreated hospital wastewater. Hence, infection prevention and control implementation at each hospital is mandatory.

One of the persistence concerns due to the existence of ESBL-producing bacteria in water bodies are related to the transmission of conjugative plasmids, which additionally carry genes resistant to sulfonamides and aminoglycosides, giving the bacteria multi-resistant patterns [45]. This phenomenon was observed in our study, where ciprofloxacin (74.6%) and SXT (73.2%) were among the ESBLs-pE. However, in this study, the most active drugs for ESBLs-pE were meropenem (94.4%), nitrofurantoin (88.7%), and piperacillin/tazobactam (87.3%).

During sample collection in this study, we noticed the selected hospitals had neither WWTP nor wastewater stabilization pond except in TASH, where a nonfunctional waste stabilization pond was observed. Since the present study detected a high proportion of pathogenic, resistant and ESBLs-pE, indicating a higher probability of potential risk of microbial pollution of water bodies in the community, hence accelerate spreading of resistant microorganisms into the community.

### Recommendation

The high occurrence of MDR and ESBLs-pE in the present study from hospital effluent may have a severe consequence on public health. These bacteria can carry several genetic determinants which can be transmitted to another bacterium, including pathogens. This indicates a need for a highly committed and consistently hygienic treatment of effluent (implementation of final disinfection procedure to minimize the microbial burden) at each respective ward and units to inhibit the transmission of the antibiotic resistant to another enteric pathogenic bacterium. Since all selected hospitals for this study purpose didn’t have either wastewater treatment plant or a functional wastewater stabilization pond; therefore, every concerned body should take urgent measures to minimize the devastating outcome from the discharge of hospital wastewater into the community drainages without getting appropriate treatment. We suggest additional studies to take place on the molecular epidemiology of ESBLs producing Enterobacteriaceae and their effects on patient recovery and health care burden in Addis Ababa, Ethiopia.

## Data Availability

The current study data sets used for analysis can be obtained from the corresponding author through email (hadalexlab@gmail.com) on reasonable request.

## Abbreviations

ALERT: All African leprosy and tuberculosis rehabilitation training center
AMR: Anti-microbial resistance
ATCC: American type culture collection
CDT: Combination disk test method
CFU: Colony forming unit
CLSI: Clinical and Laboratory Standard Institute
CTX-M: Cefotaximases Munichin
DDST: Double disk synergy test
ESBL: Extended spectrum beta-lactamase
ESBLs-pE: ESBLs producing Enterobacteriaceae
ICU: Intensive care unit
MDR: Multi-drug resistance
MDR TB Ward: Multidrug resistant tuberculosis ward
MHA: Mueller Hinton agar
MIIRH: Menelik ΙΙ referral hospital
SHV: Sulfhydryl variable
SPHHMMC: St. Paul’s Hospital Millennium Medical College
TASH: Tikur Anbessa Specialized Hospital
TEM: Temoneira
UTIs: Urinary tract infections
WWTP: Wastewater treatment plant
Y12HMC: Yekatit 12 hospital medical college
